# QRS pattern and improvement in right and left ventricular function after cardiac resynchronization therapy: a radionuclide study

**DOI:** 10.1186/1471-2261-12-27

**Published:** 2012-04-11

**Authors:** Giulia Domenichini, Haran Burri, Cinzia Valzania, Gilberto Gavaruzzi, Francesco Fallani, Mauro Biffi, Henri Sunthorn, Igor Diemberger, Cristian Martignani, Huberdine Foulkes, Eric Fleury, Giuseppe Boriani

**Affiliations:** 1 Institute of Cardiology, University of Bologna, Via Massarenti 9, Bologna 40138, Italy; 2 Cardiology Service, University Hospital of Geneva, Geneva, Switzerland; 3 Department of Nuclear Medicine, S. Orsola-Malpighi Hospital, Bologna, Italy

**Keywords:** Cardiac resynchronization therapy, Left ventricular ejection fraction, Right ventricular ejection fraction, Dyssynchrony, Nuclear angiography, QRS morphology

## Abstract

**Background:**

Predicting response to cardiac resynchronization therapy (CRT) remains a challenge. We evaluated the role of baseline QRS pattern to predict response in terms of improvement in biventricular ejection fraction (EF).

**Methods:**

Consecutive patients (pts) undergoing CRT implantation underwent radionuclide angiography at baseline and at mid-term follow-up. The relationship between baseline QRS pattern and mechanical dyssynchrony using phase analysis was evaluated. Changes in left and right ventricular EF (LVEF and RVEF) were analyzed with regard to baseline QRS pattern.

**Results:**

We enrolled 56 pts, 32 with left bundle branch block (LBBB), 4 with right bundle branch block (RBBB) and 20 with non-specific intraventricular conduction disturbance (IVCD). A total of 48 pts completed follow-up. LBBB pts had significantly greater improvement in LVEF compared to RBBB or non-specific IVCD pts (+9.6 ± 10.9% vs. +2.6 ± 7.6%, p = 0.003). Response (defined as ≥ 5% increase in LVEF) was observed in 68% of LBBB vs. 24% of non-specific IVCD pts (p = 0.006). None of the RBBB pts were responders. RVEF was significantly improved in LBBB (+5.0 ± 9.0%, p = 0.007), but not in non-specific IVCD and RBBB pts (+0.4 ± 5.8%, p = 0.76). At multivariate analysis, LBBB was the only predictor of LVEF response (OR, 7.45; 95% CI 1.80-30.94; p = 0.006), but not QRS duration or extent of mechanical dyssynchrony.

**Conclusions:**

Presence of a LBBB is a marker of a positive response to CRT in terms of biventricular improvement. Pts with non-LBBB pattern show significantly less benefit from CRT than those with LBBB.

## Background

Cardiac resynchronization therapy (CRT) reduces morbidity and mortality in patients (pts) with congestive heart failure [1-5] improving clinical status [1-3] and favoring ventricular reverse remodeling [2,5-7]. However clinical and/or echocardiographic response is present in only 50-70% of CRT pts [3,8], suggesting that the link between standard criteria for CRT and expected response is often weak. Several parameters of electrical and mechanical dyssynchrony have been proposed to improve pt selection, even though QRS duration is currently the only recommended parameter [9,10]. In CRT pts a baseline left bundle branch block (LBBB) has been demonstrated to be associated with a more favorable prognosis in terms of freedom from death or major cardiovascular events, and with a more left ventricular (LV) reverse remodeling compared to a baseline right bundle branch block (RBBB) [11,12]. Likewise in the RAFT trial [13] LBBB pts showed more benefit from CRT in terms of death or hospitalization for heart failure compared to pts with RBBB, non-specific intraventricular conduction disturbance (IVCD) or paced QRS at baseline. Furthermore, in a secondary analysis of the MADIT-CRT trial [14] a significant reduction in the risk of heart failure or death has been reported in LBBB pts within the CRT plus defibrillator (CRT-D) group. However, if the role of baseline LBBB in terms of prognosis and LV function during CRT seems to be established, there is lack of data regarding its effects on right ventricular (RV) function. Likewise, few data are available on the impact of baseline RBBB or non-specific IVCD patterns on biventricular function during CRT.

In our study we investigated the relationship between baseline QRS pattern and biventricular mechanical dyssynchrony and we evaluated the role of baseline QRS morphology to predict CRT response in terms of improvement in biventricular ejection fraction (EF). Radionuclide angiography with phase analysis was used to evaluate mechanical dyssynchrony and to measure LVEF and RVEF.

## Methods

### Patient population

We enrolled 28 pts undergoing CRT device implantation at the Cardiology Institute, University Hospital of Bologna (inclusion period: January 2007- July 2009), and 28 pts implanted at the Cardiology Service, University Hospital of Geneva (inclusion period: September 2002- December 2004). According to current guidelines [9], pts had to be in New York Heart Association (NYHA) class III or IV, with LVEF ≤ 35% and with QRS duration ≥ 120 ms. All pts were in sinus rhythm at implantation and under optimal pharmacological treatment.

A control group of 25 subjects without cardiovascular disease and with normal electrocardiogram (ECG), matched for age and sex with the study group, was evaluated to define the cut-off of inter and intraventricular dyssynchrony of phase analysis parameters.

The local institutional Ethics Committees (*Ethics Committee of the S.Orsola-Malpighi Hospital of Bologna *and *Clinical Ethics Committee of the Geneva University Hospitals*) approved the study protocol, and all patients provided a written informed consent for participation.

### Device implantation

All device leads were placed transvenously. The RV lead was positioned at the mid-septum or at the apex at the discretion of the implanting physician. The LV lead was positioned via the coronary sinus, targetting lateral or posterolateral veins. An echocardiographic-guided optimization of CRT programming was performed after implantation according to conventional clinical practice.

### Baseline electrocardiograms

A surface 12-lead ECG was recorded before implantation at speed of 25 mm/s and at 10 mm/mV gain. The QRS interval was measured from its first deflection to its end at the widest QRS complex. The QRS patterns were classified as LBBB or RBBB or non-specific IVCD according to the "AHA/ACCF/HRS Recommendations for the Standardization and Interpretation of the Electrocardiogram" [15]. Left axis deviation was defined as a QRS axis leftward of -30° [15].

### Radionuclide angiography

LVEF and RVEF were measured by radionuclide angiography at baseline, during spontaneous rhythm, and at mid-term follow-up (between 3 and 6 month from device implantation), during biventricular pacing. The response to CRT was defined by an increase in LVEF of ≥ 5% at follow-up end [16]. Radionuclide angiography was performed as previously described [17,18], using a gamma camera Prism 2000 XP- Philips in Bologna and a gamma camera ADAC-Philips in Geneva. A blood sample was drawn to label red blood cells with 1 GBq of 99mTechnetium. The ECG was monitored continuously for R-wave gating, with elimination of extrasystolic and post-extrasystolic cycles. Multigated equilibrium blood pool planar scintigrams at 32 frames/cycle (200-250 Kcounts/frame in a 128 × 128 matrix) were acquired until the number of counts was at least 6 × 10^6 ^in the "best septal separation" left anterior oblique view that provided optimal RV and LV discrimination. A background-corrected, time-activity curve was constructed by a semi-automated edge-detection method with a variable region of interest, verified visually and modified manually if necessary. LVEF and RVEF were computed on the basis of relative end-diastolic and end-systolic counts.

Inter- and intraventricular dyssynchrony were obtained by phase analysis, as previously reported [19]. The images acquired for measuring LVEF and RVEF were digitally processed to display the "phase" of each pixel overlying the ECG-gated equilibrium blood pool. The computer assigned a phase angle (between 0 and 360°) to each pixel of the image. A phase histogram was constructed, corresponding to the sequence of ventricular contraction during the cardiac cycle, with color codes corresponding to different regions of the ventricles. Each ventricle was analyzed separately, with calculation of the mean and standard deviation (SD) of the phase histogram. Interventricular delay was assessed by the absolute difference between the mean phase angles of each ventricle, whereas intraventricular delay was represented by the SD of the phase histogram for that ventricle. Both inter and intraventricular delays were expressed in angles (°).

### Statistical analysis

Statistical analyses were performed using SPSS software (SPSS Inc., Chicago, IL, USA). Continuous variables showing normal distribution according to Kolmogorov-Smirnov test were compared using paired and unpaired Student's t-tests for related and unrelated groups, respectively. Data showing non-Gaussian distribution were processed using the Mann-Whitney test. Correlations between quantitative variables were examined using Pearson's test. Fisher's exact test was used for evaluating categorical variables. Logistic regression analysis was performed to evaluate the relationship between baseline clinical parameters and CRT response. Values were expressed as mean ± SD or median and interquartile range (IQR) in case of non-Gaussian distribution. Odds ratios (OR) were presented with 95% confidence intervals (CIs). A P-value of less than 0.05 was considered statistically significant.

## Results

We enrolled a total of 56 pts, 42 males, age 66 ± 11 years (see Table 1). Thirty-four pts received a CRT-D device. LV lead position was lateral/posterolateral in 49 pts (88%) and anterolateral in 7 pts (12%); RV lead was positioned at the mid-septum in 11 pts (20%). There were no significant differences between groups as regards the position of LV and RV leads. A total of 48 pts completed follow-up after a median of 5.5 (3.5-7.1) months. Two pts (1 LBBB and 1 RBBB) died for non-cardiac causes and 6 pts dropped out (3 LBBB and 3 non-specific IVCD).

**Table 1:** Demographics of the patient population

Patient demographics (*n *= 56)	
Age (years)	66 ± 11
Males	42 (75%)
Aetiology of heart failure	
Ischaemic	22 (39%)
Non-ischaemic	34 (61%)
NYHA class	
III	49 (88%)
IV	7 (12%)
Paroxysmal AF	5 (9%)
Hypertension	30 (54%)
Diabetes	19 (34%)
Chronic kidney disease	14 (25%)
QRS morphology	
LBBB	32 (57%)
RBBB	4 (7%)
Non-specific IVCD	20 (36%)
QRS duration (ms)	140 (120-160)
QRS ≥ 150 ms	27 (48%)
LVEF (%)	22 ± 6
RVEF (%)	36 ± 10
Traitement	
ACE-I/ARBs	52 (93%)
Beta-blockers	39 (70%)
Diuretics	50 (89%)

NYHA class, New York Heart Association class; AF, atrial fibrillation; LBBB, left bundle branch block; RBBB, right bundle branch block; IVCD, intraventricular conduction disturbance; LVEF, left ventricular ejection fraction; RVEF, right ventricular ejection fraction; ACE-Is, angiotensin-converting enzyme inhibitors; ARBs, angiotensin II receptor blockers

### Baseline QRS pattern and mechanical dyssynchrony

In the control group (age 63 ± 4 years, 13 males, LVEF 62 ± 6%, RVEF 48 ± 5%), intraventricular delay for the left ventricle was 10.9° (8.2-16.1°) and 16.9° (12.3-19.6°) for the right ventricle. The interventricular delay was 5.0° (3.0-11.5°). Values greater than the 95th percentile of the distribution curve (after rounding to a multiple of 5°) were considered as dyssynchronous. Therefore, we defined intraventricular dyssynchrony as SD values > 20° for the LV phase and as SD values > 25° for the RV phase. Interventricular dyssynchrony was defined as values > 15°.

Table 2 shows the baseline clinical characteristics of the study population according to QRS pattern. The QRS duration was significantly longer in the LBBB group compared to the non-specific IVCD group. Furthermore, non-ischemic aetiology of heart failure was prevalent in the LBBB pts and male sex was more represented in the non-specific IVCD group.

**Table 2:** Baseline characteristics according to QRS pattern

	LBBB (*n *= 32)	RBBB (*n *= 4)	Non-specific IVCD (*n *= 20)
Age (years)	66 ± 11	65 ± 9	67 ± 10
Males	21 (66%)	2 (50%)	19 (95%)*
Aetiology of heart failure			
Ischaemic	9 (28%)	3 (75%)	10 (50%)
Non-ischaemic	23 (72%)	1 (25%)	10 (50%)
NYHA class			
III	28 (88%)	4 (100%)	17 (85%)
IV	4 (12%)	0 (0%)	3 (15%)
QRS duration (ms)	160 (140-180)	140 (125-170)	120 (120-130)^§^
QRS ≥ 150 ms	22 (69%)	1 (25%)	4 (20%)^†^
LEFT axis	15 (47%)	3 (75%)	10 (50%)
LVEF (%)	23 ± 6	25 ± 6	21 ± 7
RVEF (%)	37 ± 11	38 ± 9	34 ± 8

*p = 0.018 vs LBBB; ^§^p < 0.001 vs LBBB; ^†^p = 0.001 vs LBBB

LBBB, left bundle branch block; RBBB, right bundle branch block; IVCD, intraventricular conduction disturbance; NYHA class, New York Heart Association class; LVEF, left ventricular ejection fraction; RVEF, right ventricular ejection fraction

The comparisons of mechanical dyssynchrony according to QRS pattern and QRS duration are shown in Table 3. The differences in dyssynchrony between the groups did not reach statistical significance, although comparisons are limited by small sample size and data dispersion.

**Table 3:** Comparison of mechanical dyssynchrony according to QRS pattern and QRS duration

	Control group	LBBB	RBBB	Non-specific IVCD	QRS < 150 ms	QRS ≥ 150 ms
	*(n = 25)*	*(n = 32)*	*(n = 4)*	*(n = 20)*	*(n = 29)*	*(n = 27)*
SD of LV mean phase (°)	10.9 (8.2-16.1)	42.3 (24.7-77.9)*	35.4 (28.8-39.2)*	43.4 (36.0-59.6)*	36.9 (27.0-50.4)*	48.4 (29.5-78.1)*
SD of RV mean phase (°)	16.9 (12.3-19.6)	27.2 (18.8-38.8)*	20.4 (11.9-34.9)	25.5 (17.8-36.0)*	26.1 (18.8-35.9)*	25.5 (17.4-39.8)*
Interventricular delay (°)	5.0 (3.0-11.5)	21.0 (8.5-34.8)*	17.5 (4.0-40.0)	14.0 (6.5-25.3)*	19.0 (6.5-28.0)*	21.0 (8.0-28.0)*

Intra LV dyssynchrony		28 (88%)	4 (100%)	20 (100%)	26 (90%)	25 (93%)
Intra RV dyssynchrony		18 (56%)	2 (50%)	10 (50%)	16 (55%)	14 (52%)
Interventricular dyssynchrony		22 (69%)	2 (50%)	10 (50%)	16 (55%)	17 (63%)

*p < 0.01 vs control group

LBBB, left bundle branch block; RBBB, right bundle branch block; IVCD, intraventricular conduction disturbance; SD, standard deviation; LV, left ventricular; RV, right ventricular

### Baseline QRS pattern and changes in biventricular ejection fraction at follow-up

**Figure 1:**
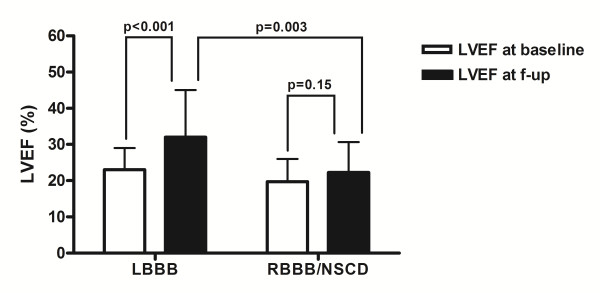
**Baseline QRS pattern and changes in left ventricular ejection fraction (LVEF) at follow-up**. LBBB, left bundle branch block; RBBB, right bundle branch block; NSCD, non-specific intraventricular conduction disturbance.

**Figure 2:**
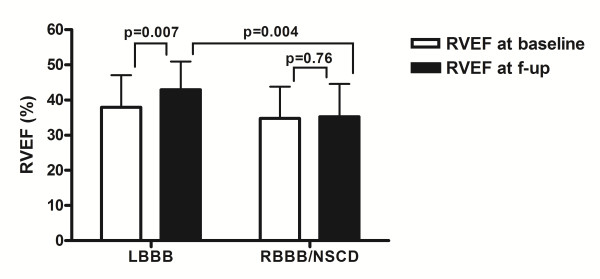
**Baseline QRS pattern and changes in right ventricular ejection fraction (RVEF) at follow-up**. LBBB, left bundle branch block; RBBB, right bundle branch block; NSCD, non-specific intraventricular conduction disturbance.

CRT response was observed in 68% of LBBB vs. 24% of non-specific IVCD pts (p = 0.006). None of the RBBB pts were responders. Pts with LBBB had significantly greater improvement in LVEF compared to those with RBBB and non-specific IVCD (+9.6 ± 10.9% vs. +2.6 ± 7.6%, p = 0.003) (see Figure 1). RVEF was significantly improved in LBBB pts (+5.0 ± 9.0%, p = 0.007), but not in non-specific IVCD and RBBB pts (+0.4 ± 5.8%, p = 0.76) (see Figure 2). No significant correlation was found between increase in LVEF and increase in RVEF in LBBB pts (*r *= 0.22, p = 0.25).

### Reproducibility of radionuclide angiography

LVEF and RVEF and LV and RV mean phase angle reproducibility were assessed in independent readings, done by the same operator, of the same nuclear examination.

We found that the difference between two independent measurements, expressed in absolute terms, was 0.49 ± 2.47% (centre A) and 0.56 ± 1.58% (centre B) for LVEF; 0.10 ± 3.5% (centre A) and 0.65 ± 6.1% (center B) for RVEF; 2.20 ± 8.84° (centre A) and 3.39 ± 4.63° (centre B) for LV mean phase angle; 2.01 ± 7.31° (centre A) and 2.28 ± 9.91° (centre B) for RV mean phase angle. These data indicate that the differences between two independent measurements of RVEF or LVEF or mean phase angles were negligible in absolute terms. Furthermore no significant differences were found between the two centres.

### Baseline clinical characteristics predicting response to CRT

Baseline LBBB showed a significantly higher prevalence in CRT responders compared to RBBB and non-specific IVCD (p = 0.001) (see Table 4). Furthermore, CRT responders tended to have greater QRS duration and also tended to have more frequently cardiomyopathy of non-ischemic origin, though significant differences were not present. Finally, mechanical dyssynchrony parameters did not differ significantly between the two groups. At multivariate analysis, LBBB was the only predictor of CRT response (OR, 7.45; 95% CI 1.80-30.94; p = 0.006) (see Table 5).

**Table 4:** Baseline clinical characteristics according to CRT response at follow-up

	Responders (*n *= 23)	Non Responders (*n *= 25)	*p*
Age (years)	67 ± 11	65 ± 11	0.64
Males	15 (65%)	21 (84%)	0.19
Aetiology of heart failure			
Ischaemic	6 (26%)	11 (44%)	0.24
Non-ischaemic	17 (74%)	14 (56%)	
NYHA class			
III	21 (87%)	23 (92%)	0.67
IV	3 (13%)	2 (8%)	
QRS duration (ms)	150 (120-160)	140 (120-180)	0.77
QRS ≥ 150 ms	12 (52%)	12 (48%)	> 0.99
LEFT axis	11 (48%)	14 (56%)	0.77
QRS morphology			
LBBB	19 (83%)	9 (36%)	0.001*
RBBB	0 (0%)	3 (12%)	0.24^§^
Non-specific IVCD	4 (17%)	13 (52%)	0.017^†^
LVEF (%)	23 ± 5	20 ± 6	0.05
RVEF (%)	37 ± 8	36 ± 10	0.74
SD of LV mean phase (°)	43.0 (24.0-73.0)	43.0 (35.5-64.5)	0.48
SD of RV mean phase (°)	28.0 (19.0-40.0)	26.0 (17.5-36.5)	0.40
Interventricular delay (°)	21.0 (8.0-38.0)	20.0 (8.5-27.5)	0.56
Intra LV dyssynchrony	20 (87%)	24 (96%)	0.34
Intra RV dyssynchrony	16 (70%)	13 (52%)	0.25
Interventricular dyssynchrony	16 (70%)	15 (60%)	0.56

*LBBB vs. RBBB and non-specific IVCD; §RBBB vs. LBBB and non-specific IVCD; † non-specific IVCD vs. LBBB and RBBB.

NYHA class, New York Heart Association class; LBBB, left bundle branch block; RBBB, right bundle branch block; IVCD, intraventricular conduction disturbance; LVEF, left ventricular ejection fraction; RVEF, right ventricular ejection fraction; SD, standard deviation; LV, left ventricular; RV, right ventricular

**Table 5:** Baseline clinical characteristics predicting response to CRT

	Univariate analysis OR (95%CI)	*p*	Multivariate analysis OR (95%CI)	*p*
Males	0.36 (0.09-1.41)	0.14		
Age (years)	1.01 (0.96-1.01)	0.63		
IHD	0.45 (0.13-1.52)	0.20		
Diabetes	0.42 (0.12-1.49)	0.18		
Hypertension	1.18 (0.38-3.67)	0.77		
Chronic kidney disease	1.41 (0.37-5.45)	0.62		
LBBB	8.44 (2.18-32.66)	0.002	7.47 (1.80-30.94)	0.006
RBBB	*n.a*.	*n.a*.		
Non-specific IVCD	0.19 (0.05-0.74)	0.016		
QRS duration (ms)	1.00 (0.98-1.02)	0.90		
QRS ≥ 150 ms	1.18 (0.38-3.67)	0.77		
LBBB and QRS ≥ 150 ms	2.81 (0.85-9.28)	0.09		
Left axis	0.72 (0.23-2.25)	0.57		
LVEF (%)	1.11 (1.00-1.23)	0.06		
RVEF (%)	1.01 (0.95-1.08)	0.70		
Intra LV dyssynchrony	0.28 (0.03-2.88)	0.28		
Intra RV dyssynchrony	2.11 (0.65-6.90)	0.22		
Interventricular dyssynchrony	1.52 (0.46-5.04)	0.49		

IHD, ischemic heart disease; LBBB, left bundle branch block; RBBB, right bundle branch block; IVCD, intraventricular conduction disturbance; LVEF, left ventricular ejection fraction; RVEF, right ventricular ejection fraction; LV, left ventricular; RV, right ventricular

## Discussion

The main findings of our study may be summarized as follows: 1. A baseline LBBB morphology is a marker of a positive response to CRT in terms of improvement not only in LVEF but also in RVEF; 2. Pts with a baseline RBBB or an IVCD pattern seem to derive less benefit from CRT compared to those with LBBB.

Our study included pts with both non-ischemic as well as ischemic cardiomyopathy and assessed the response to CRT in terms of both right and left ventricular function. In the literature limited attention has been paid to RV function, and this can be easily explained by the limitation of echocardiographic approaches. In pts with idiopathic dilated cardiomyopathy, Fauchier *et al. *[20] demonstrated a relationship between QRS morphology and inter- and intraventricular delays at phase analysis. In particular, a high intra-LV dyssynchrony was associated with LBBB and left axis deviation. In our study, despite the lack of correlation between QRS morphology and degree of mechanical dyssynchrony, LBBB pts showed a significant improvement in biventricular EF compared to RBBB and IVCD pts. Fantoni *et al. *[21], using a three-dimensional non-fluoroscopic electroanatomic contact mapping system, documented a similar degree of LV activation delay in LBBB and RBBB heart failure pts. However RBBB pts presented also a delayed activation of anterior and lateral RV regions, that was absent in pts with LBBB. These data may suggest a more complex electromechanical profile in heart failure RBBB pts, explaining a poor response to CRT and reflecting at the same time the need to identify specific selection criteria.

Unfortunately, the prevalence of RBBB and non-specific IVCD is very low in the major CRT randomized trials (about 10% for RBBB) [4,22], therefore the available data for these groups of pts are limited. In the analysis of pooled data from the MIRACLE and Contak CD trials [23] the LVEF or maximal oxygen consumption did not improve significantly at 6 months follow-up in RBBB pts randomly assigned to CRT. Aldestein *et al. *[12] evaluated 636 consecutive pts undergoing to CRT implantation with LBBB, RBBB, or paced QRS at baseline. RBBB pts had low rates of symptomatic and echocardiographic response and the survival free from orthotopic heart transplantation or ventricular assist device placement was significantly worse compared to LBBB pts. In another study of Rickard *et al. *[24] pts with RBBB and non specific-IVCD had less reverse remodeling and symptomatic benefit from CRT compared with those with a native LBBB. Likewise in the CRT-D arm of the MADIT-CRT trial [25] LBBB pts showed a significantly higher reduction in LV volumes and a significant increase in LVEF compared to non-LBBB pts. Our study is in agreement with these results. Furthermore, we demonstrated that LBBB pts also had a significant improvement in RVEF (although the magnitude of the response was less than for LVEF), whereas pts with non-LBBB had no significant improvement in RVEF. The mechanisms by which LBBB pattern promotes improvement in RVEF in response to CRT are unclear [26], but may result in part from a reduction in right ventricular afterload with improvement in left-sided pump function.

### Study limitations

The small RBBB sample size was the main limitation of our study, reducing the statistical value of results for this specific group of pts. However, as previously reported, the low prevalence of RBBB candidates to CRT reflects a common condition encountered in clinical practice as well as in most trials during pts selection for CRT. In this context, further prospective studies are needed to investigate the relationship between electromechanical dyssynchrony and CRT response in RBBB pts in order to improve pts selection and optimize the device system capabilities in these pts.

## Conclusion

In this prospective study we evaluated the relationship between QRS pattern, mechanical dyssynchrony and CRT response. LBBB is a significant marker of a positive response to CRT, irrespective of QRS duration, in terms of improvement not only in LVEF but also in RVEF. Pts with non-LBBB pattern seem to benefit less from CRT than those with LBBB. Further evaluations to clarify the mechanisms allowing RVEF improvement in LBBB pts are required.

## Competing interests

The authors declare that they have no competing interests.

## Authors' contributions

GD: data collection, data analysis/interpretation, statistics, drafting article. HB: concept/design, data analysis/interpretation, drafting article, critical revision of article, approval of article. CV: data collection, data analysis/interpretation. GG: data collection, data analysis/interpretation. FF: data collection, data analysis/interpretation. MB: data collection, data analysis/interpretation. HS: data collection, data analysis/interpretation. ID: data collection, data analysis/interpretation. CM: data collection, data analysis/interpretation. HF: data collection. EF: data collection. GB: concept/design, data analysis/interpretation, drafting article, critical revision of article, approval of article. All authors read and approved the final manuscript.

## Pre-publication history

The pre-publication history for this paper can be accessed here:

http://www.biomedcentral.com/1471-2261/12/27/prepub
